# Social cognition in chronic migraine with medication overuse: a cross-sectional study on different aspects of mentalization and social relationships

**DOI:** 10.1186/s10194-023-01578-1

**Published:** 2023-04-28

**Authors:** Sara Bottiroli, Alessia Rosi, Grazia Sances, Marta Allena, Roberto De Icco, Serena Lecce, Tomaso Vecchi, Cristina Tassorelli, Elena Cavallini

**Affiliations:** 1 Giustino Fortunato University, Benevento, Italy; 2 IRCCS Mondino Foundation, Pavia, Italy; 3 Department of Brain and Behavioral Sciences, University of Pavia, Pavia, Italy

**Keywords:** Chronic migraine, Medication overuse headache, Theory of mind, Socio-cognitive dimensions

## Abstract

**Background:**

Social cognition refers to all mental operations to decipher information needed in social interactions. Here we aimed to outline the socio-cognitive profile of Chronic Migraine with Medication Overuse (CM + MO), given they are recognized to be at risk of socio-cognitive difficulties. Given the multidimensionality of this construct, we considered: (1) socio-cognitive abilities, (2) socio-cognitive beliefs, (3) alexithymia and autism traits, and (4) social relationships.

**Methods:**

Seventy-one patients suffering from CM + MO, 61 from episodic migraine (EM), and 80 healthy controls (HC) were assessed with a comprehensive battery: (1) the Faux Pas test (FP), the Strange Stories task (SS), the Reading Mind in the Eyes test (RMET), (2) the Troms*ø* Social Intelligence Scale, (3) the Toronto Alexithymia Scale, the Autism Spectrum Quotient, (4) the Lubben Social Network Scale, the Friendship Scale.

**Results:**

CM + MO: (1) performed similar to EM but worse than HC in the FP and SS, while they were worse than EM and HC in the RMET; (2) were similar to EM and HC in social intelligence; (3) had more alexithymic/autistic traits than EM and HC; (4) reported higher levels of contact with their family members but felt little support from the people around them than HC.

**Conclusions:**

CM + MO results characterized by a profile of compromised socio-cognitive abilities that affects different dimensions. These findings may have a relevant role in multiple fields related to chronic headache: from the assessment to the management.

**Supplementary Information:**

The online version contains supplementary material available at 10.1186/s10194-023-01578-1.

## Introduction

Migraine is among the most common and disabling neurological disorders [1]. In many sufferers, attacks recur less than 15 days/month (episodic migraine – EM), however in a small but significant portion of patients the disease evolves into a chronic pattern (chronic migraine—CM). Transition from EM to CM often occurs in association with a Medication Overuse (CM + MO) [2]. Current evidence suggests that psychological aspects may play a relevant role in CM, in particular in case of MO. Indeed, the existing association between CM + MO and psychopathologies [3-8]—which may have an impact on the outcome of treatment [9-12]- is well established. Moreover, most of the CM + MO patients tend to present alexithymia [13-15], i.e., a cognitive-affective disturbance affecting how individuals experience and express their internal states [16, 17].

Besides psychopathological comorbidities, additional factors have been emerging as linked to CM + MO. The biopsychosocial model suggests the existence of a complex interrelationship among biological, psychological, and psychosocial vulnerabilities [18]. Thus, the diversity in migraine expression results from the interplay among these factors, able to shape perceptions and response to disease [19].

When referring to psychosocial vulnerabilities, it is important to include also all those activity limitations and social-interactions restrictions being impacted by chronic pain [20]. Recent evidence showed an association between migraine and social cognitive functioning [21-23], whose decline could be responsible for dysfunctional social behaviors and personal distress. The term “social cognition” is a wide construct that refers to all mental operations that allow to decipher information about the intentions and affective states of social partners [24]. One of these social cognitive abilities is the capacity to infer one’s own and others’ mental states such as desires, emotions, and beliefs [25] (Theory of Mind, ToM).

In pediatric migraine [26-30], it has been showed an association between physical sickness and mental representation and way to think [31, 32], resulting in difficulties in expressing emotional states [33] and higher level of alexithymia [34, 35]. Less evidence is available in adult migraine. Bouteloup [21] comparing severe EM and CM patients with healthy controls (HC) found difficulties in the clinical populations in social and emotional cognition, which was explained as due to high alexithymic levels. Raimo [23] explored the neuropsychological correlates of ToM and found that CM patients had evident difficulties in the cognitive dimension involved in inferring other’s mental states. Romozzi [22] compared CM + MO, EM, and HC in complex emotion recognition, knowledge about one’s own and other person’s mental states, and alexithymic levels and found an impairment in all considered dimensions in CM + MO patients. Therefore, it appears that a dysfunction in social cognitive abilities may represent a critical characteristic of CM/CM + MO. The limited number of studies in adult samples calls for additional and more fine-grained investigations.

Regarding the ToM construct, one of the main distinctions refers to the *affective* (i.e., attributions regarding others' emotional mental states) and *cognitive* (i.e., knowledge of others' mental states such as beliefs, thoughts and intentions) components [36, 37]. One might expect these two components to be altered in CM + MO [21-23], but to what extent has not been explored yet. As socio-cognitive functioning also includes other aspects, such as difficulty in expressing one’s emotional states [33], what one thinks about one’s abilities in social situations [38], and the perception of social engagement [39], personal beliefs about social interactions represent a further field of investigation.

The present study aimed to characterize the socio-cognitive profile of CM + MO patients compared to EM and HC individuals by investigating four different aspects of social cognition: (a) affective and cognitive components of socio-cognitive abilities; (b) beliefs about one’s social cognitive functioning; (c) alexithymia and autism traits; (d) levels of social relationships experienced in everyday life. As in different clinical conditions [40, 41] association between social cognitive abilities and psychological distress and reduced quality of life (QoL) emerged, we also investigated this relationship. Our hypothesis is that CM + MO patients are characterized by a specific socio-cognitive profile that differentiate them from both EM and HC.

## Methods

### Participants

This is a cross-sectional case–control study conducted at the Headache Science and Neurorehabilitation Center (a tertiary referral center) of the Mondino Foundation in Pavia, Italy. We enrolled consecutive patients with stable (i.e., migraine duration ≥ 10 years) EM and patients with CM + MO. An expert neurologist verified the eligibility criteria during the recruitment process based on history, headache diaries, and neurological evaluation. A group of HC was enrolled as well and were community-based volunteers recruited from the general population. All participants completed a vocabulary test (drawn by Primary Mental Abilities test [42]) as a cognitive control variable of semantic knowledge. The study was approved by the Ethics Committee of San Matteo Hospital (Pavia, Italy) and written informed consent was obtained from all patients. Inclusion and exclusion criteria are reported in Table 1. The protocol followed the Strengthening the Reporting of Observational Studies in Epidemiology (STROBE) reporting guidelines for cross-sectional investigations [43].

**Table 1:** Demographic and clinical characteristics of patients enrolled in the study

	**CM + MO** ***n* = 71** **mean ± SD**	**EM** ***n* = 61** **mean ± SD**	**HC** ***n* = 80** **mean ± SD**
Inclusion criteria	a) age > 18, < 65 years b) ICHD-III criteria for CM + MO^2^	a) age > 18, < 65 years b) ICHD-III criteria for EM^2^ c) migraine duration ≥ 10 years d) no history of CM	age > 18, < 65 years
Exclusion criteria	a) dementia b) psychosis c) mental retardation	a) dementia b) psychosis c) mental retardation	a) personal or family history of migraine b) dementia c) psychosis d) mental retardation
Age	43.89 ± 8.94^*^	41.54 ± 10.43	38.00 ± 11.88^*^
Gender (female %)	83%	84%	66%
Years of education	13.29 ± 3.48	14.39 ± 2.76	14.00 ± 3.13
Vocabulary	46.18 ± 2.44	45.95 ± 2.79	45.76 ± 2.48
Age at onset (years)	14.35 ± 8.04	16.44 ± 8.29	-
Duration of chronic headache (months)	68.29 ± 67.32	-	-
Days with headache per month	26.22 ± 4.76	6.48 ± 2.85	-
Days with intake per month	24.20 ± 5.71	-	-
Drug doses per month	41.01 ± 28.62	-	-
Class of overused acute drug		-	-
NSAIDs	18%	-	-
Triptans	24%	-	-
Combination	50%	-	-
Multiple drug classes	8%	-	-
Patients on prophylaxis treatment	38%	-	-
Amitriptyline	22%	-	-
Propranolol	10%		
Venlafaxine	3%	-	-
Topiramate	6%	-	-

*Note*: *CM* + *MO* Chronic Migraine with Medication Overuse, *EM* Episodic Migraine, *HC* Healthy Control; Vocabulary: range score 0–50; *NSAIDs* nonsteroidal anti-inflammatory drugs. *Denotes significant differences between CM + MO and HC, *F*(2,209) = 5.97, *p* = 0.03

### Procedure

Each consultation was performed by a headache expert that diagnosed the headache type, collected socio-demographic data, and migraine characteristics. Participants also underwent an interview with a psychologist during which they filled a series of tests and scales (a detailed description is reported below and in Table 2).

**Table 2:** Description of tests used in the study

Test	Subtests/subscales	Description	Outcome
Faux Pas test (FP)	Cognitive	False belief question which tested whether participants understood the false beliefs of who committed the faux pas	Socio-cognitive abilities
	Affective	Affective question which tested the emphatic understanding of how the person in the story would feel	
Strange Stories task (SS)	Mentalistic stories	Ability to understand people’s mental states (i.e., cognitive components of ToM) and described situations in which two or more characters interact with each other in social contexts	Socio-cognitive abilities
	Physical stories	Ability to make inferences on physical (rather than mental) events and required the integration of information between sentences and inference from implicit information	
Reading Mind in the Eyes Test (RMET)	Experimental test	Ability to infer affective mental states of the person in the photograph (i.e. affective components of ToM)	Socio-cognitive abilities
	Control test	Judge the gender of the person in the photograph	
Troms*ø* Social Intelligence Scale (TSIS)	Social Information processing	Own beliefs to understand and predict other people’s behavior and feelings	Socio-cognitive beliefs
	Social skills	Own beliefs to understand basic communication skills in social situations	
	Social Awareness	Own beliefs to behave in accordance with social situations	
Toronto Alexithymia Scale (TAS-20)	Factor 1 (F1)	Items are referred to the ability to identify feelings and distinguish them from bodily sensations	Alexithymia traits
	Factor 2 (F2)	Items related to a concrete thinking style	
	Factor 3 (F3)	Items concerning the ability to express emotion and fantasy (daydreaming)	
Autism Spectrum Quotient (AQ)	Social Skill	Items assessing social skills in social situation	Autism traits
Lubben Social Network Scale-Revised (LSNS-R)	Relatives	Each subscale (six items) containing items that measure size, closeness, and frequency of contact within respondent’s social network	Social relationships
	Friends		
Friendship Scale (FS)	Total score	Overall sum scores assessing perceived social support	Social relationships
	Levels of social support	Classification of responders into five levels of perceived social support: (1) very socially isolated, (2) isolated, (3) some social support, (4) socially connected, and (5) highly socially connected. Due to low frequencies, the first two and the last two categories were combined to create three levels of perceived social support: low (scores 0–15), moderate (scores 16–18), and high (scores 19–24) social support	
Hospital Anxiety and Depression Scale (HADS)	Anxiety	Items assessing the severity of anxiety symptoms	Psychological assessment
	Depression	Items assessing the severity of depression symptoms	
World Health Organization Quality of Life Brief Version (WHOQOL-BREF)	-	Items assessing levels of quality of life	Quality of life assessment

### Socio-cognitive measures

#### Socio-cognitive abilities

Faux pas test (FP [44]; Italian version [45]) consisted of 4 short stories containing a faux pas assessing both the cognitive (i.e., False Beliefs) and the affective components of ToM.

Strange Stories task (SS [46]) consisted of 4 Mentalistic stories and 4 Physical stories. After having read the stories, participants were asked to answer the test question explaining the reasons why the characters behaved as they did.

Reading Mind in the Eyes Test (RMET [47]) consisted of 36 black-and-white photographs of the eye-region of the face, depicting a specific mental state. Participants were required to choose which one among four adjectives best described what the person in the photograph was feeling (experimental test) and to judge his/her gender (control test).

#### Socio-cognitive beliefs

Troms*ø* Social Intelligence Scale (TSIS [48]; Italian version [49]) was a self-report inventory including 21 items with three subscales: Social Information processing, Social Skills, and Social Awareness.

#### Alexithymia and autism traits

The presence of alexithymia was investigated using the 20-item version of the Toronto Alexithymia Scale (TAS-20; Italian version [50]) consisting of three factors: Factor 1, Factor 2, and Factor 3.

The presence of autistic traits was investigated using the Social Skills subscale of the Autism Spectrum Quotient (AQ [51]; Italian validation [52]).

#### Social relationships

Lubben Social Network Scale-Revised (LSNS-R [53]) was used to measure people’s social relationships with relatives and friends.

The Friendship Scale (FS [54]) was a 6-item scale assessing perceived social support.

### Psychological and quality of life assessment

Levels of depression and anxiety were evaluated using the Hospital Anxiety and Depression Scale (HADS [55]) whereas QoL was assessed by using the World Health Organization Quality of Life Brief Version (WHOQOL-BREF [56]).

### Statistical analyses

Performance on the FP was considered as primary outcome given it allows to assess both cognitive and affective components of socio-cognitive abilities. The sample size was calculated on this outcome, showing that a total of 206 participants was needed to discover an effect size of 0.25 with 0.90 statistical power and α = 0.05 in mixed ANOVA.

Data are presented as means ± SD for continuous data and n/% for frequency data. The differences between groups were examined with χ^2^ tests for categorical variables and analysis of variance (ANOVA) for quantitative variables. We first checked the equivalence of groups on demographic variables. We then examined group differences on socio-cognitive variables and psychological assessment with one-way and mixed Analysis of Covariance (ANCOVA). Then, we ran correlation analyses to examine the relationship between socio-cognitive and psychological variables for every separate diagnostic category. Two binary logistic regression models were performed to identify those socio-cognitive and psychological variables which best differentiate CM + MO patients from the other two groups (first model: CM + MO vs HC; second model: CM + MO vs EM). The criterion for predictors’ inclusion in the regression models was the existence of significant group differences at the level of *p* < 0.05 at univariate analysis.

## Results

### Study population

Two hundred and twelve subjects were enrolled (see descriptives in Table 1). We observed an older age in CM + MO than HC, whereas EM did not differ from the other two groups. No other significant differences between groups resulted in the demographic variables.

### Socio-cognitive measures

Descriptives and statistics for socio-cognitive measures are reported in Table 3.

**Table 3:** Means and standard deviations for the socio-cognitive and psychological characteristics of the CM + MO, EM, and HC groups

	**Range**	**CM + MO** ***n* = 71** **mean ± SD**	**EM** ***n* = 61** **mean ± SD**	**HC** ***n* = 80** **mean ± SD**	***F*(2,209*)***	***p***	***η***^***2***^_***p***_
	**Socio-cognitive abilities **						
***Faux Pas test (FP)***							
FP Cognitive ^+^	0–4	2.95 ± 0.99	2.82 ± 0.85^e^	3.26 ± 0.82^e^	4.80	0.009	0.04
FP Affective ^+^	0–4	2.77 ± 0.96^d^	2.87 ± 0.92^e^	3.39 ± 0.83^d,e^	8.65	< 0.001	0.08
***Strange Stories task (SS)***							
SS Mentalistic	0–8	5.58 ± 1.77^d^	5.92 ± 1.83^e^	6.65 ± 1.20^d,e^	8.91	< 0.001	0.08
SS Physical	0–8	6.56 ± 1.10	6.11 ± 1.43	6.26 ± 1.35	2.08	0.127	0.02
***Reading the Mind in the Eyes Test (RMET)***							
RMET Experimental	0–36	20.77 ± 4.38^c,d^	23.62 ± 3.92^c^	24.00 ± 3.16^d^	15.31	< 0.001	0.13
RMET Control	0–36	35.44 ± 1.28	35.20 ± 0.83	35.14 ± 0.74	1.90	0.152	0.02
	**Socio-cognitive beliefs**						
***Tromsø Social Intelligence Scale (TSIS)***							
TSIS Total	21–147	94.59 ± 17.91	96.94 ± 14.69	98.35 ± 13.66	0.745	0.476	0.01
TSIS Social Information	7–49	29.65 ± 6.89	31.15 ± 7.80	31.90 ± 7.17	1.88	0.155	0.02
TSIS Social Skill	7–49	33.20 ± 8.98	32.96 ± 9.90	35.72 ± 7.81	2.02	0.135	0.02
TSIS Social Awareness	7–49	31.75 ± 6.91	32.84 ± 6.62	30.72 ± 6.63	2.36	0.097	0.02
	**Alexithymia and Autism traits**						
***Toronto Alexithymia Scale (TAS-20)***							
TAS-20 Total	20–100	47.62 ± 10.12^c,d^	42.61 ± 11.20^c^	42.58 ± 10.20^d^	5.85	0.003	0.05
TAS-20 Factor 1 (F1)	7–35	19.66 ± 5.02^c,d^	17.28 ± 5.23^c^	17.55 ± 3.90^d^	6.01	0.003	0.05
TAS-20 Factor 2 (F2)	5–25	13.79 ± 3.66^d^	12.70 ± 4.23	12.06 ± 3.74^d^	3.93	0.021	0.04
TAS-20 Factor 3 (F3)	8–40	14.17 ± 4.20	12.62 ± 4.14	13.27 ± 4.42	3.93	0.093	0.04
***Autism Quotient (AQ)***	0–10	3.59 ± 2.52^c,d^	2.26 ± 1.55^c^	2.32 ± 2.08^d^	8.34	< 0.001	0.07
	**Social relationships**						
***Lubben Social Network Scale – Revised (LSNS-R)***							
LSNS-R Total	0–30	38.79 ± 8.40	38.46 ± 7.31	37.31 ± 8.17	2.15	0.118	0.02
LSNS-R Family	0–15	20.66 ± 3.96^d^	20.16 ± 4.78	18.47 ± 4.96^d^	5.15	0.007	0.05
LSNS-R Friends	0–15	18.13 ± 6.05	18.29 ± 4.31	18.84 ± 5.33	0.05	0.948	0.01
***Friendship Scale (FS)***							
FS Total	0–24	17.62 ± 5.77	18.39 ± 4.83	19.22 ± 3.94	1.44	0.238	0.01
FS social support (%)					11.80^a^	0.019^a^	0.24^b^
*Low social support*		33.8	21.3	15			
*Moderate social support*		8.5	19.7	25			
*High social support*		57.7	59	60			
	**Psychological and Quality of Life assessment**						
***Hospital Anxiety and Depression Scale (HADS)***							
HADS Anxiety	0–21	7.89 ± 4.12	7.33 ± 4.66	6.80 ± 4.21	0.94	0.391	0.01
HADS Depression	0–21	7.61 ± 5.05^c,d^	5.64 ± 4.19^c^	5.49 ± 3.67^d^	4.25	0.016	0.016
***The World Health Organization Quality of Life (WHOQOL-BREF)***	8–40	25.97 ± 5.33^c,d^	28.68 ± 4.96^c^	29.97 ± 4.99^d^	11.38	< 0.001	0.11

*Note*: *CM* + *MO* Chronic Migraine with Medication Overuse, *EM* Episodic Migraine, *HC* Healthy Control; + p values referring to one-way ANCOVA performed on cognitive and affective components with Group (CM + MO vs. EM vs. HC) as between-subject variable, covarying for age. We found a main effect of Group for both the cognitive, F(2,208) = 4.80, *p* = 0.009, and the affective components, F(2,208) = 8.65, *p* < 0.001. ^a^Chi square test values; ^b^Phi correlation as a measure of effect size; ^c^significant differences between CM + MO and EM; ^d^significant differences between CM + MO and HC; ^e^significant differences between EM and HC

#### Socio-cognitive abilities

Looking at the FP, the analyses showed group differences for both the cognitive and the affective components. For the cognitive component, the HC group outperformed the group of EM, and for the affective component HC outperformed the CM + MO and EM groups.

For the SS, the analyses revealed group differences for the Mentalistic condition, with HC group outperforming the CM + MO and EM groups, and not for the Physical condition.

For the RMET, results showed group differences in the Experimental condition, with the HC and the EM groups outperforming the CM + MO group, and not for the Control condition. Detailed analyses are reported in Supplementary Table 1.

#### Socio-cognitive beliefs

Results did not show significant group differences nor in the TSIS total score neither in the subscales, *p*s ≥ 0.097.

#### Alexithymia and autism traits

Concerning alexithymia, analyses revealed significant group differences on the TAS-20 total score, F1, and F2 subscales. Post-hoc tests revealed that for the TAS-20 total score and F1 subscale, the CM + MOH group showed higher scores compared to the EM patient and HC group. For the F2 subscale, CM + MO group reported significantly higher scores compared to the HC group. No significant group differences emerged for the F3 subscale.

For the AQ test, results showed significant group differences: the CM + MO group had significantly higher scores compared to the EM patients and HC group.

#### Social relationships

Concerning the LSNS-R, analyses did not report significant group differences for the total score and friends’ subscale. Significant group differences emerged in the family subscale where CM + MO group showed significantly higher scores than the HC group.

No group differences emerged in the FS total score. However, looking at the frequency of participants falling in the category of low, moderate, and high social support, χ^2^ revealed significant group differences. The frequency of CM + MO patients falling in the low social support category was higher than the other two groups and the frequency of HC subjects falling into the moderate social support category was higher compared to CM + MO and EM patients.

### Psychological and QoL assessment

Descriptives and statistics for Psychological and QoL assessment are reported in Table 3. Significant group differences were found in the HADS depression subscale and not in the HADS anxiety subscale, where the CM + MO group had significantly higher depression scores than the EM patient and HC group. Concerning QoL, group differences emerged in the WHOQOL-BREF where CM + MO group had significantly lower scores compared to the EM patient and HC group.

### Correlation analyses

To verify whether socio-cognitive abilities were associated with the other variables in which group differences were found, we ran correlation analyses separately for each group (see Table 4). For the CM + MO group, the Mentalistic SS correlated negatively with the AQ and the HADS depression subscale, and positively with the FS categories and the WHOQOL-BREF. Regarding the EM group, the Mentalistic SS correlated negatively with the AQ and HADS depression subscale, and positively with the LSNS-R family subscale. For the HC group, the FP Cognitive component correlated positively with the LSNS-R family subscale. No other correlations were found.

**Table 4:** Correlation between socio-cognitive abilities and other variables in which we found group differences, separately for each group

	**FP** **Cognitive **	**FP ** **Affective **	**SS ** **Mentalistic**	**RMET Experimental**
**CM + MO group**				
TAS-20 Factor 1	-0.17	-0.01	-0.13	0.12
TAS-20 Factor 2	-0.05	-0.05	-0.06	-0.16
AQ	-0.04	0.03	-0.45***	0.16
LSNS-R—Family	0.14	-0.01	0.23	0.08
FS social support	0.18	-0.11	0.25*	-0.19
HADS—Depression	-0.17	-0.07	-0.53***	0.03
WHQOL-BREF	0.56	0.29	0.32**	-0.23
**EM group**				
TAS-20 Factor 1	-0.21	-0.22	-0.10	-0.06
TAS-20 Factor 2	-0.06	-0.06	-0.12	-0.05
AQ	-0.09	0.21	-0.30*	0.18
LSNS-R—Family	-0.03	0.21	0.35**	-0.09
FS social support	0.01	0.11	0.01	0.49
HADS—Depression	-0.05	0.01	-0.34**	-0.10
WHQOL-BREF	0.06	0.09	0.11	-0.04
**HC group**				
TAS-20 Factor 1	0.05	0.15	-0.21	0.17
TAS-20 Factor 2	-0.07	-0.01	-0.21	-0.07
AQ	-0.07	-0.01	-0.08	-0.16
LSNS-R—Family	0.27*	0.12	0.05	-0.09
FS social support	0.05	-0.06	-0.01	0.01
HADS—Depression	-0.09	-0.02	0.04	-0.06
WHQOL-BREF	-0.14	-0.17	0.07	-0.01

*Note: CM* + *MO* Chronic Migraine with Medication Overuse, *EM* Episodic Migraine, *HC* Healthy Control, *FP* Faux Pas test, *SS* Strange Stories task, *RMET* Reading Mind in the Eyes Test, *TAS-20* Toronto Alexithymia Scale, *AQ* Autism Quotient, *LSNS-R* Lubben Social Network Scale – Revised, *FS* Friendship Scale, *HADS* Hospital Anxiety and Depression Scale, *WHQOL-BREF* World Health Organization Quality of Life.  The variable FS social support is a categorical variable coded as 1 = low social support, 2 = moderate social support, 3 = high social support

***p < .001; **p < .01; *p < .05

### Predictors of CM + MO

The binary logistic regression models’ results are summarized in Table 5.

**Table 5:** Regression coefficients (β) and odds (OR) and 95% CI Corresponding to the Binary Logistic Regression Model

Category	*β*	*SE*	*p*	OR (95% CI)
*CM* + *MO vs. HC*				
SS Mentalistic	-0.202	0.189	0.285	0.82 (0.56–1.18)
FP Cognitive	-0.119	0.319	0.708	0.89 (0.48–1.66)
**FP Affective **	**-0.893**	**0.319**	**0.005**	**0.41 (0.22–0.76)**
**RMET Experimental**	**-0.330**	**0.083**	** < 0.001**	**0.72 (0.61–0.85)**
TAS-20 F1	0.079	0.077	0.303	1.08 (0.93–1.26)
TAS-20 F2	0.046	0.087	0.600	1.05 (0.88–1.24)
FS – High vs. Low	-0.015	0.754	0.984	0.98 (0.22–4.32)
**FS – High vs. Moderate**	**-1.955**	**0.859**	**0.023**	**0.14 (0.03–0.76)**
AQ	0.231	0.153	0.130	1.26 (0.93–1.70)
**LSNS-R Family**	**0.122**	**0.056**	**0.031**	**1.13 (1.01–1.26)**
HADS-Depression	-0.139	0.099	0.162	0.87 (0.72–1.06)
**WHOQoL**	**-0.316**	**0.085**	** < 0.001**	**0.79 (0.62–0.86)**
*CM* + *MO vs. EM*				
SS Mentalistic	0.153	0.166	0.357	1.16 (0.84–1.61)
FP Cognitive	0.2402	0.295	0.173	1.49 (0.84–1.08)
FP Affective	-0.515	0.303	0.089	0.60 (0.33–1.08)
**RMET Experimental**	**-0.247**	**0.069**	** < 0.001**	**0.78 (0.68–0.89)**
TAS-20 F1	0.088	0.058	0.130	1.09 (0.97–1.22)
TAS-20 F2	-0.034	0.077	0.667	0.97 (0.83–1.13)
FS – High vs. Low	-0.038	0.660	0.954	0.96 (0.26–3.51)
FS – High vs. Moderate	-1.206	0.877	0.169	0.30 (0.05–1.67)
**AQ**	**0.408**	**0.152**	**0.007**	**1.50 (1.12–2.03)**
LSNS-R Family	0.096	0.060	0.111	1.10 (0.98–1.24)
HADS-Depression	-0.095	0.089	0.287	1.10 (0.98–1.24)
**WHOQoL-BREF**	**-0.137**	**0.068**	**0.044**	**0.87 (0.76–0.99)**

*Note:* *CM+MO* Chronic Migraine with Medication Overuse, *EM *Episodic Migraine, *HC *Healthy Control, *SS* Strange Stories, *FP* Faux Pas, *RMET *Reading the Mind in the Eyes Test, *TAS-20* Toronto Alexithymia Scale, *F1* Factor 1, *F2* Factor 2, *FS* Friendship Scale, *AQ* Autism Quotient,* LSNS-R* Lubben Social Network Scale – Revised, *HADS* Hospital Anxiety and Depression Scale, *WHOQoL-BREF* The World Health Organization Quality of Life Brief Version. First regression model: χ^2^(12) = 100.68, *p* < .001; Second regression model: χ^2^(12) = 42.98, *p* < .001. Significant odds ratio (OR) in bold

For the first model, significant predictors that increased the odds to be in the CM + MO group than the HC group were: lower performance in the FP Affective component and the RMET experimental condition, lower prevalence of moderate social support in the LS, higher scores in the family subscale of the LSNS-R, and lower scores in the WHOQOL-BREF. This logistic regression model was statistically significant and it explained 65% (Nagelkerke R^2^) of the variance. This model correctly classified 90% of the CM + MO patients, and 90% of the HC group.

In the second model, significant predictors that increased the odds to be in the CM + MO group than the EM group were lower performance in the RMET Experimental condition, lower scores in the WHOQOL-BREF, and higher score in the AQ. The model was statistically significant and it explained 42% (Nagelkerke R^2^) of the variance. This model correctly classified 87.1% of the CM + MO patients, and 62.2% of the EM patients.

## Discussion

The present study aimed to outline the socio-cognitive profile of CM + MO. For what concerns socio-cognitive abilities, we had two interesting findings. First, when looking at the differences across socio-cognitive components, a more evident impairment resulted in the affective dimension for CM + MO patients. Second, the HC outperformed both migraine groups, which resulted almost always – with the only exception of the RMET – similar to each other. Such a similitude between CM + MO and EM populations is not surprising if we consider that they represent two different expressions of the same disease. In addition, our EM population had a long history of disease, which was serious enough to push them to seek care in a tertiary referral center. Thus, it is possible that a long exposure to a disabling pain condition may have affected patients’ ability to infer others’ mental states. However, although the two migraine groups were similar in several aspects, a more marked impairment resulted for those with CM + MO with respect to EM in the affective dimension of socio-cognitive abilities. Such results are partially in contrast with Raimo [23] that showed slightly greater difficulties of CM for the cognitive component than the affective one. However, in that study [23], CM patients were compared only with HC, and not with EM as we did. We believe that these affective difficulties should be considered in light of the greater levels of alexithymic and autism traits characterizing CM + MO. Autism research has shown that difficulties in identifying and describing feelings are associated to impairments in recognizing verbal and non-verbal emotional expressions [57-59], and difficulties in experiencing and understanding emotions [60].

Regarding socio-cognitive beliefs, a new topic in migraine research, we found no differences between groups in participants’ perception of how successfully they manage social relationships [61]. Our data for the three groups are in line with the norms of TSIS [48], suggesting that all participants had positive beliefs about their social abilities. If we consider this result in light of the group differences found in the performances, it could be argued that CM + MO patients did not have a clear awareness of their socio-cognitive competencies.

Regarding alexithymia, our results, as found in the literature [13-15], showed an impairment in CM + MO. It corroborates the idea that alexithymia represents a risk factor increasing susceptibility to disease [62]. This is also supported by the differences across groups we found in the autism traits, since it exists a strong association between alexithymic and autism traits, which could be explained by shared characteristics [63, 64]. It is important to consider that these aspects are critical for successful social interactions in everyday life [65]. Interestingly, the CM + MO patients differed significantly from EM and HC individuals in a specific alexithymic facet, which is the difficulty in identifying feelings and distinguishing between feelings and the bodily sensations. Indeed, individuals with high levels of alexithymic traits experience difficulties also for what concerns their non-affective interoceptive state [66]. The inaccurate identification of their bodily sensations [60] could determine a delay in seeking medical treatment [67] and favor substance use disorders [68]. In conclusion, more marked group differences in this alexithymic trait in disfavor of the CM + MO group seems to corroborate previous findings on non-affective interoceptive deficits [66], and may explain MO.

Data regarding social relationships are particularly interesting. On the one hand, we found that the CM + MO group reported higher levels of contact with their family members than the HC did, highlighting chronic patients’ need to maximize the interactions with their relatives. On the other hand, these same patients perceived that they were little supported from the people around them. These findings, although they might seem at odds, actually further highlight patients’ social difficulties. The topic of social relationships in migraine is much debated. There is evidence reporting that migraine patients are less satisfied with social support than the general population [69-71]. Belot [71] found that patients with severe migraine judged the social support they received worse than the general population did. Others [72] reported that poor social support and loneliness in CM were associated with a tendency to MO. Our research group [73] has also shown that both CM and EM patients felt emotionally lonely and less supported than HC after the COVID-19 outbreak. By contrast, it has been shown that headache sufferers had slightly more social support from their families than non-headaches sufferers [74]. Hence, it is difficult to draw definitive conclusions given the heterogeneity of the clinic populations [69-72, 74]. Moreover, these social difficulties should be interpreted at the light of the stigma experienced by CM + MO patients, perceiving a sort of discrimination against their health condition [75-77]. Indeed, our results suggest that CM + MO patients place importance on tangible support from their family members, although they do not feel much satisfied with the support received from social networks [69, 78, 79].

As the importance of socio-cognitive components in defining patients’ profile, we also searched for variables that may predict CM + MO. Patients scored lower in two affective socio-cognitive abilities tests, reported lower prevalence of moderate vs high social support, higher levels of contacts with family members, and lower QoL when compared with HC. It may be that chronic pain has affected social interactions, making patients less adept at inferring others’ mental states. It may also be that long-standing migraine has resulted in less interest in others’ mental and affective states, which in turn may have caused a reduction of social interactions. Both interpretations can be taken as true, in a circular relationship, according to the biopsychosocial model [19].

A similar pattern, albeit less pronounced, was found in the second logistic regression analysis: CM + MO was predicted by lower performances in the ability to infer others’ affective state from looking at the eyes, higher levels of autism traits, and lower QoL than EM. Even if the two migraine groups resulted similar in many socio-cognitive aspects, CM + MO patients were found to be more affected and with lower well-being due to their clinical condition, with negative consequences on understanding others. These factors may act together and predispose the development of this complex clinical condition.

Our study is not without limitations. First, we have not included a screening scale of cognitive functioning and we did not control for prophylaxis treatment that may have impact on cognition, such as topiramate [80]. However, it should be noted that we assessed participants’ semantic knowledge as a cognitive control variable that was previously associated with better performance in socio-cognitive abilities [81, 82]. Moreover, the fact itself that CM + MO showed a differentiated pattern of performance in socio-cognitive abilities, being more impaired in the affective that in the cognitive dimension, could allow us to exclude a generalized deficit due to pharmacological treatments. Second, we did not collect a comprehensive psychopathological assessment. However, we believe that it is important to focus on additional components beside psychopathologies [3-8]. Third, since this was a cross-sectional study, we are unable to define the causal trajectory involving socio-cognitive components in CM + MO. Fourth, because we did not include a group of CM patients without MO, we cannot definitively conclude that the impairment of social cognition we found is a critical feature of CM + MO rather than CM alone. Sixth, the data collection procedure did not reflect the general migraine population, as participants were recruited from a tertiary referral center. Therefore, the transferability of these results to general practice will require confirmation on larger subgroups of patients, in multicenter studies, and with different cultures.

## Conclusions

Among the causal aspects that could determine the transition to chronic migraine, we believe that a critical role should be attributed to socio-cognitive factors. Specifically, our results showed that socio-cognitive abilities, traits of alexithymia and autism, and a particular pattern of social relationships are associated with CM + MO. From a theoretical point of view, our data add an important element to the identification of risk factors for the development of this disabling condition. From a practical point of view, they have a multifold relevance: i) they provide tangible data on the social impairments associated to the condition of EM and, even more severely, of CM + MO and ii) they underscore the importance of optimizing the management of patients through a thorough preliminary assessment of their socio-cognitive profile; ii) they call for adequate public health interventions to prevent the evolution of EM into CM + MO.

## Supplementary Information

**Additional file 1.** STROBE Statement—checklist of items that should be included in reports of observational studies.

**Additional file 2: Table S1.** Correlation between socio-cognitive abilities and other variables in which we found group differences, separately for each group.

## Data Availability

The datasets presented in this study can be found in online repositories. The names of the repository/repositories and accession number(s) can be found below: [Zenodo; Reservation 10.5281zenodo.7713963].

## Abbreviations

ANCOVA: Analysis of Covariance
ANOVA: Analysis of Variance
AQ: Autism Spectrum Quotient
CM: Chronic Migraine
EM: Episodic Migraine
FP: Faux Pas test
FS: Friendship Scale
HADS: Anxiety and Depression Scale
HC: Healthy Control
LSNS-R: Lubben Social Network Scale-Revised
MO: Medication Overuse
NSAIDs: nonsteroidal anti-inflammatory drugs
QoL: Quality of Life
RMET: Reading Mind in the Eyes Test
SS: Strange Stories task
STROBE: Strengthening the Reporting of Observational Studies in Epidemiology
TAS-20: Toronto Alexithymia Scale
ToM: Theory of Mind
TSIS: Troms*ø* Social Intelligence Scale
WHOQOL-BREF: World Health Organization Quality of Life Brief Version
