# Supplementary material for: Social cognition in chronic migraine with medication overuse: a cross-sectional study on different aspects of mentalization and social relationships
Source: J Headache Pain. 2023 Apr 28;24(1):47. doi: 10.1186/s10194-023-01578-1 (PMC10139829; doi:10.1186/s10194-023-01578-1)
Supplement: Supplementary file 2 — Additional file 2: Table S1. Correlation between socio-cognitive abilities and other variables in which we found group differences, separately for each group. [file 10194_2023_1578_MOESM2_ESM.docx]

Supplementary Material

**Table S1.** Correlation between socio-cognitive abilities and other variables in which we found group differences, separately for each group.

|  | **FP**  **Cognitive** | **FP**  **Affective** | **SS**  **Mentalistic** | **RMET Experimental** |
| --- | --- | --- | --- | --- |
| **CM+MO group** |  |  |  |  |
| TAS-20 Factor 1 | -0.17 | -0.01 | -0.13 | 0.12 |
| TAS-20 Factor 2 | -0.05 | -0.05 | -0.06 | -0.16 |
| AQ | -0.04 | 0.03 | -0.45*** | 0.16 |
| LSNS-R - Family | 0.14 | -0.01 | 0.23 | 0.08 |
| FS social support | 0.18 | -0.11 | 0.25* | -0.19 |
| HADS - Depression | -0.17 | -0.07 | -0.53*** | 0.03 |
| WHQOL-BREF | 0.56 | 0.29 | 0.32** | -0.23 |
| **EM group** |  |  |  |  |
| TAS-20 Factor 1 | -0.21 | -0.22 | -0.10 | -0.06 |
| TAS-20 Factor 2 | -0.06 | -0.06 | -0.12 | -0.05 |
| AQ | -0.09 | 0.21 | -0.30* | 0.18 |
| LSNS-R - Family | -0.03 | 0.21 | 0.35** | -0.09 |
| FS social support | 0.01 | 0.11 | 0.01 | 0.49 |
| HADS - Depression | -0.05 | 0.01 | -0.34** | -0.10 |
| WHQOL-BREF | 0.06 | 0.09 | 0.11 | -0.04 |
| **HC group** |  |  |  |  |
| TAS-20 Factor 1 | 0.05 | 0.15 | -0.21 | 0.17 |
| TAS-20 Factor 2 | -0.07 | -0.01 | -0.21 | -0.07 |
| AQ | -0.07 | -0.01 | -0.08 | -0.16 |
| LSNS-R - Family | 0.27* | 0.12 | 0.05 | -0.09 |
| FS social support | 0.05 | -0.06 | -0.01 | 0.01 |
| HADS - Depression | -0.09 | -0.02 | 0.04 | -0.06 |
| WHQOL-BREF | -0.14 | -0.17 | 0.07 | -0.01 |
| Note: FP=Faux pas test; SS= Strange Stories task; RMET=Reading Mind in the Eyes test; TAS-20= Toronto Alexithymia Scale; AQ=Autism Quotient; LSNS-R=Lubben Social Network Scale – Revised; FS=Friendship Scale; HADS=Hospital Anxiety and Depression Scale; WHQOL-BERF= World Health Organization Quality of Life. The variable FS social support is a categorical variable coded as 1= low social support, 2=moderate social support, 3=high social support.  ***p<.001; **p<.01; *p<.05 | | | | |
